# From sequence analysis of DPP-4 to molecular docking based searching of its inhibitors

**DOI:** 10.6026/97320630016444

**Published:** 2020-06-30

**Authors:** Awad Saeed Alsamghan, Afaf S Alwabli, Mohammed Abadi, Safar A Alsaleem, Dalia Mohammed Anbari, Amani Saleh Alomari, Othman Alzahrani, Qamre Alam, Mohammed Tarique

**Affiliations:** 1 Department of Family and Community Medicine, College of Medicine, King Khalid University, Abha, KSA-61421; 2 Department of Biological Sciences, Faculty of Science, King Abdulaziz University, Jeddah 21589, Kingdom of Saudi Arabia; 3 Department of Family and Community Medicine, College of Medicine, King Khalid University, Abha, KSA-61421; 4 Department of Family and Community Medicine, College of Medicine, King Khalid University, Abha, KSA-61421; 5 Department of Biochemistry, Faculty of Science, King Abdulaziz University, Jeddah 21589, Kingdom of Saudi Arabia; 6 Department of Biochemistry, Faculty of Science, King Abdulaziz University, Jeddah 21589, Kingdom of Saudi Arabia; 7 Department of Biology, Faculty of Sciences, University of Tabuk, Tabuk 71491, Kingdom of Saudi Arabia; 8 Genome and Biotechnology Unit, University of Tabuk, Tabuk 71491, Kingdom of Saudi Arabia; 9 Medical Genomics Research Department, King Abdullah International Medical Research Center, King Saud bin Abdulaziz University for Health Sciences, Ministry of National Guard Health Affairs, Riyadh, Kingdom of Saudi Arabia; 10 Center for Interdisciplinary Research in Basic Sciences, Jamia Millia Islamia, Jamia Nagar, New Delhi-110025, India

**Keywords:** DPP-4, GLP-1, diabetes, docking analysis, inhibitor

## Abstract

Literature data suggests that Dipeptidyl peptidase-4 (DPP-4) is a potential target for type 2 Diabetes Mellitus. Therefore, it is of interest to identify new DPP-4 inhibitors using molecular docking analysis. We document compounds such as STOCK1N-98884, STOCK1N-98881, and STOCK1N-98866 with optimal binding features with DPP-4 from the ligand database at https://www.ibscreen.com/ for further consideration.

## Background

Insulin resistance in type 2 diabetes and related issues are known [1]. Symptoms associated with the disease include retinopathy, edema, micro aneurysms, nephropathy outlines, symmetrical fringe neuropathy influencing engine and tactile nerves of the smaller attachments [2-4]. Several models of treatments using insulin, secretagogues (sulfonylureas and incretins) and hypoglycemias (biguanides, thiazolidinediones and a-glucosidase inhibitors) are currently available [5-10]. Inhibitors of the dipeptidyl peptidase-4 (DPP-4) are linked with the activities of GLP-1 and gastric inhibitory polypeptide (GIP) [7,8]. Description of the structural models for DPP-4 is known [15-17]. Therefore, it is of interest to identify molecules to inhibit DPP-4 using molecular docking analysis.

## Materials and Methods:

### Sequence to structure modeling and docking analysis of DPP-4:

The DPP-4 protein sequence downloaded from GenBank was analyzed in a comprehensive using tools such as Clustal Omega, Pfam, Prosite, SMART, PANTHER, PHYLIP, STRING and InterProScan, molecular docking and ligand-protein analysis tools to glean valuable insights [11-21].

## Results and discussion:

**Figure 1:**
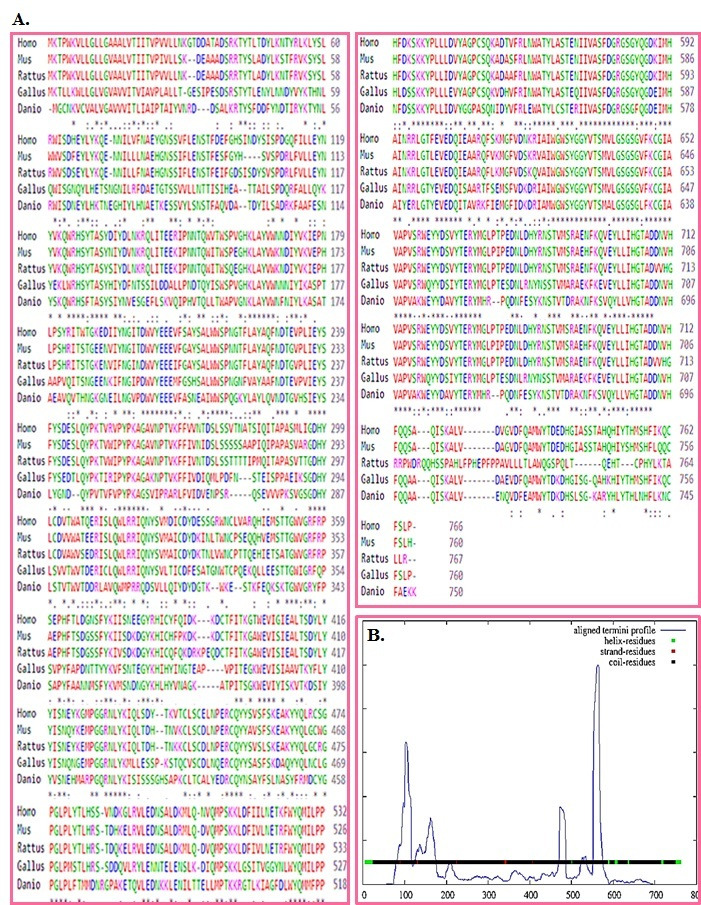
(A) MSA of DDP-4 from different organisms (Homo sapiens, Rattus norvegicus, Mus musculus, Danio rerio and Gallus gallus) (B) Secondary structure information on DDP-4

**Figure 2:**
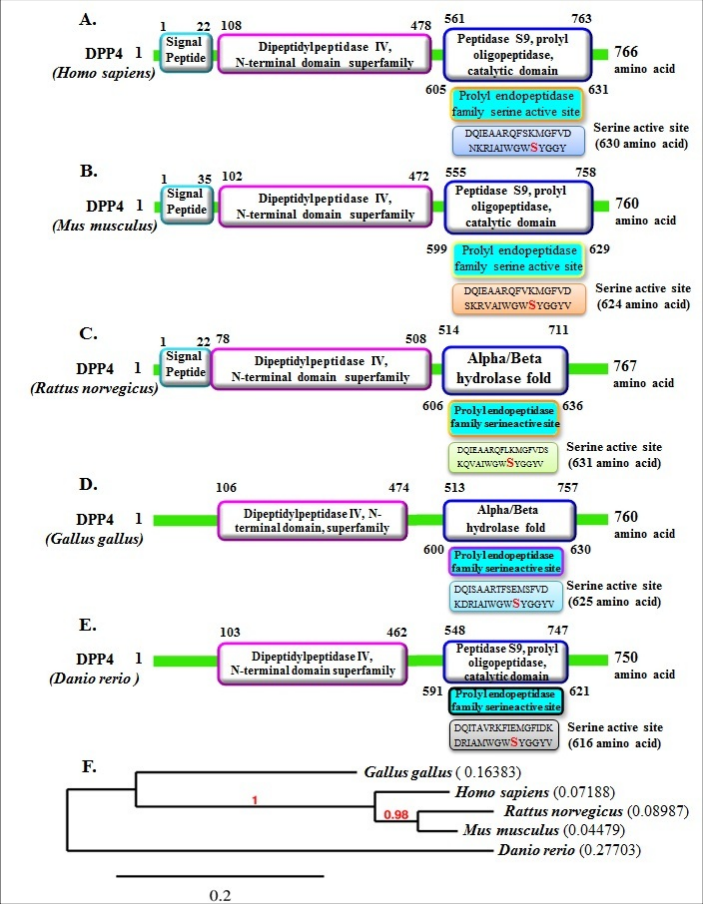
Domain and phylogeny analysis of DDP-4 in different organisms

**Figure 3:**
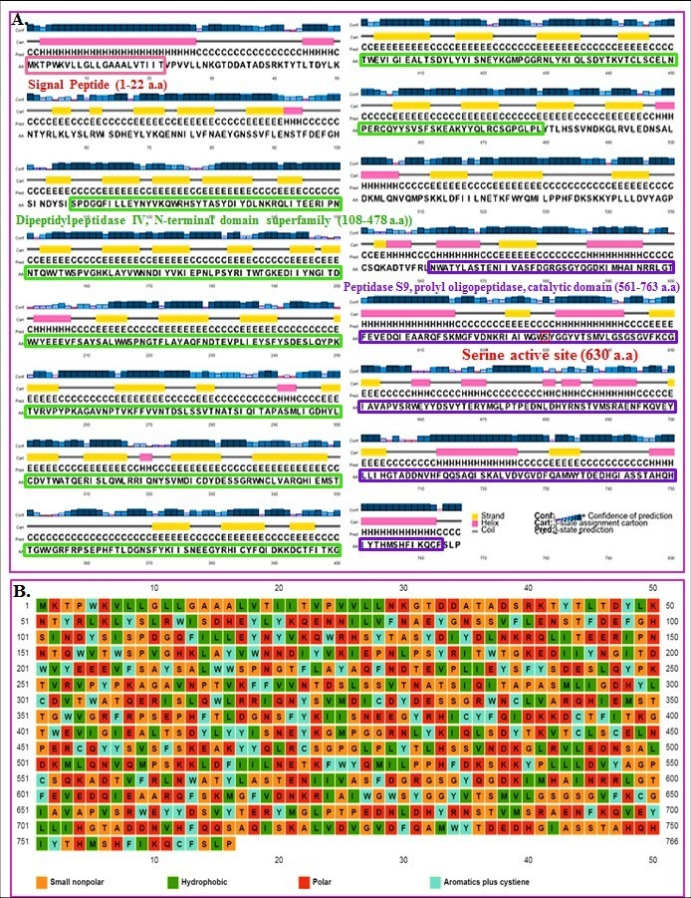
(A) Secondary structure analysis of human DDP-4 (Homo sapiens). (B) Small non-polar, hydrophobic, polar and aromatic plus cysteine residues in human DDP-4.

**Figure 4:**
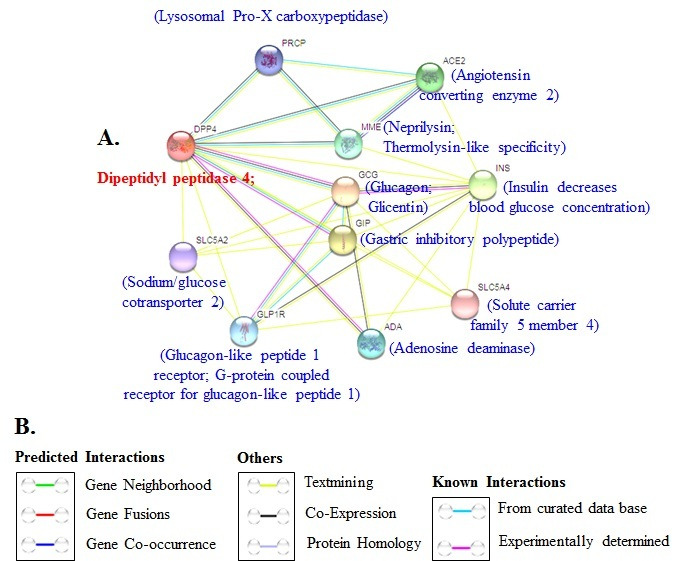
(A) Interacting proteins with DPP4 using STRING v10.database. (B) Explanation of interactions shown.

**Figure 5:**
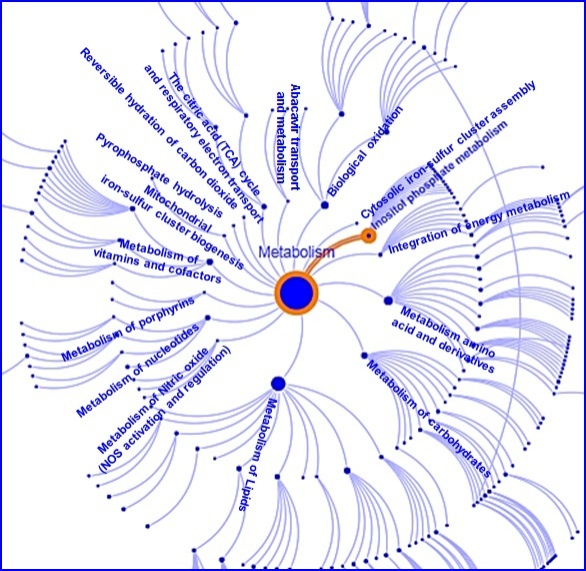
DDP-4 linked pathways.

**Figure 6:**
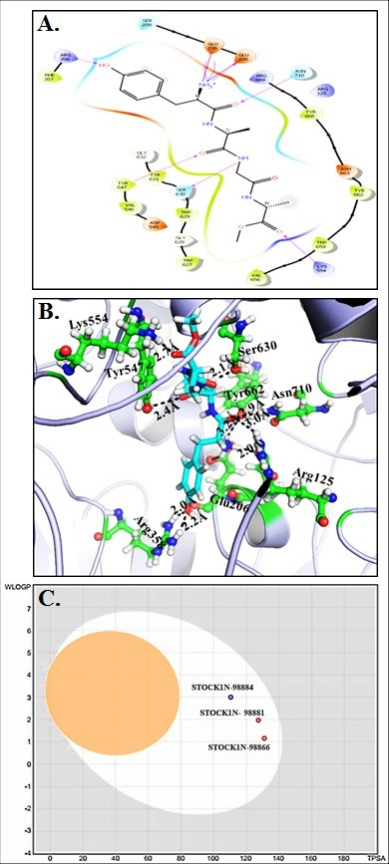
(A) Molecular docking interaction of DPP4 with STOCK1N-98884. (B) Cartoon interpretation of DPP4 with compound STOCK1N-98884. (C) Boiled-egg plot.

A comprehensive analysis of DDP-4 using sequence and structure information is highly relevant in the fight against T2DM with reference to known data in the literature. The Multiple Sequence Analysis (MSA) of DDP-4 from different organisms such as Homo sapiens (DPP4, 766 amino acid), Rattus norvegicus (DPP4, 767 amino acid), Mus musculus (DPP4, 760 amino acid), Danio rerio (DPP4, 750 amino acid) and Gallus gallus (DPP4, 760 amino acid) is given in Figure 1. Secondary structure information of DDP-4 is also shown in Figure 1. Domain and phylogeny analysis of DDP-4 in different organisms is given in Figure 2.The Secondary structure analysis of human DDP-4 along with small nonpolar, hydrophobic, polar, and aromatic plus cysteine residues in human DDP-4 is shown in Figure 3. Protein-protein interaction network linked to DDP-4 is shown in Figure 4. We further show the DDP-4 associated pathways in Figure 5. The molecular docking interaction of DPP4 with STOCK1N-98884 is given in Figure 6 and Table 1-Table 4. This information gleaned from the analysis of DDP-4 is relevant in the design and development of novel compounds in combating the disease.

## Conclusions:

We document compounds STOCK1N-98884, STOCK1N-98881, and STOCK1N-98866 from the IBS ligand database with optimal binding features with DPP-4 towards combating T2DM.

## Figures and Tables

**Table 1:** Lowest binding energy for the Ligands-Protein interaction, along with scores for various interaction types, as detected by GLIDE GScore; Glide extra precision scores (kcal/mol) Lipophilic E Vdw; Chemscore lipophilic pair term and fraction of the total protein–ligand vdw energy HBond; Hydrogen-bonding term

Compounds ID	Binding Energy MM-GBSA (kcal/mol)	GScore	Lipophilic E vdw	H-bond	Electro	Protein ligands interaction
STOCK1N-98884	-72.7837	-11.56	-2.91	-6.87	-2.01	Glu:205, Glu:206, Try :547, Ser:630 and Asn710
STOCK1N-98881	-61.2792	-10.2	-3.37	-4.44	-2.41	Arg:125, Glu:205, Glu:206, Lys:554, Trp:629 and Ser:630
STOCK1N-98866	-59.2571	-9.58	-2.46	-3.65	-3.19	Arg:125, Try :547, Lys:554 and Trp:629
Known Inhibitor						
Linagliptin	-44.1282	-6.79	-2.22	-2.61	-0.34	Try :547, Ser:630 and Asn710
Electro; Electrostatic rewards Protein ligands interaction; p–p stacking, p–cat interaction and hydrogen bond between the ligands and protein

**Table 2:** Evaluation of drug-like properties of the lead molecules by Qikprop Maestro 10.5 molecular docking suite

Molecule	QPlog Po/w (-2.0 to 6.5)	Q P log HERG (acceptable ange:above -5.0)	QPP Caco (nm/s)<25—poor>500—great	Q P log,BB(-3 to 1.2)	QPP MDCK (nm/s)	Q Plog Kp (-8.0 to -0.1)
STOCK1N-98884	-0.3	-1.056	131.328	-0.94	70.119	-2.798
STOCK1N-98881	3.376	-0.015	283.926	-0.628	485.3	-2.406
STOCK1N-98866	2.219	-3.804	143.431	-1.641	60.643	-3.179
Predicted IC50 value for blockage of HERG K+ channels; (acceptable range above -5.0) Molecule STOCK, InterBioScreen’s library (IBS), Q P log Poct; was predicted partition coefficient of octanol/gas, (8.0 to 35.0); QPP Caco, predicted apparent Caco-2 cell permeability in nm/s. Caco-2 cells is a model for the gut blood barrier (nm/s) <25-poor, >500-great. Q P log BB, predicted brain/blood partition coefficient; QPP MDCK, predicted apparent MDCK cell permeability in nm/s. MDCK cells are considered to be a good mimic for the blood–brain barrier; (nm/s) <25-poor, >500-great; Q P log KP, Predicted skin permeability; Q P log Khsa Prediction of binding to human serum albumin; (acceptable range -1.5 to 1.5)

**Table 3:** Boiled egg parameters

Molecule	MW	TPSA	XLOGP3	MLOGP	GI absorption	BBB permeant
STOCK1N-98884	430.88	159.85	-0.3	-0.66	High	No
STOCK1N-98881	624.04	158.3	2.99	0.23	Low	No
STOCK1N-98866	421.4	127.08	2.81	0.93	High	No

**Table 4:** Biological activity spectrum of compounds (Pa – Active; Pi – Inactive)

Molecule	Pa	Pi	Activity
STOCK1N-98884	1.219	0.449	Anti-diabetic
STOCK1N-98881	1.812	0.642	Anti-diabetic
STOCK1N-98866	1.121	0.318	Anti-diabetic
